# Global Oxidative Status Is Linked to Calcific Aortic Stenosis: The Differences Due to Diabetes Mellitus and the Effects of Metformin

**DOI:** 10.3390/antiox12051024

**Published:** 2023-04-28

**Authors:** Nerea Corbacho-Alonso, Elena Rodríguez-Sánchez, Tamara Sastre-Oliva, Elisa Mercado-García, Ines Perales-Sánchez, Cristina Juarez-Alia, Luis F. López-Almodovar, Luis R. Padial, Teresa Tejerina, Laura Mourino-Alvarez, Gema Ruiz-Hurtado, María G. Barderas

**Affiliations:** 1 Department of Vascular Physiopathology, Hospital Nacional de Paraplejicos, SESCAM (Servicio de Salud de Castilla-La Mancha), 45071 Toledo, Spain; 2 Department of Vascular Physiopathology, Hospital Nacional de Paraplejicos, Instituto de Investigación Sanitaria de Castilla-La Mancha (IDISCAM), 45071 Toledo, Spain; 3 Cardiorenal Translational Laboratory, Instituto de Investigación Imas12, Hospital Universitario 12 de Octubre, 28041 Madrid, Spain; 4 Cardiac Surgery, Hospital General Universitario de Toledo, SESCAM, 45007 Toledo, Spain; 5 Department of Cardiology, Hospital General Universitario de Toledo, SESCAM, 45007 Toledo, Spain; 6 Department of Pharmacology, School of Medicine, Universidad Complutense de Madrid, 28040 Madrid, Spain; 7 Centro de Investigación Biomédica en Red de Enfermedades Cardiovasculares, CIBER-CV Hospital Universitario 12 de Octubre, 28041 Madrid, Spain

**Keywords:** calcific aortic stenosis, diabetes mellitus, oxidative stress, antioxidant defense, metformin

## Abstract

Calcific aortic stenosis (CAS) and type 2 diabetes mellitus (T2DM) are related and often concomitant pathologies, accompanied by common comorbidities such as hypertension or dyslipidemia. Oxidative stress is one of the mechanisms that trigger CAS, and it can drive the vascular complications in T2DM. Metformin can inhibit oxidative stress, yet its effects have not been studied in the context of CAS. Here, we assessed the global oxidative status in plasma from patients with CAS, both alone and with T2DM (and under treatment with metformin), using multimarker scores of systemic oxidative damage (OxyScore) and antioxidant defense (AntioxyScore). The OxyScore was determined by measuring carbonyls, oxidized LDL (oxLDL), 8-hydroxy-20-deoxyguanosine (8-OHdG), and xanthine oxidase (XOD) activity. In contrast, the AntioxyScore was determined through the catalase (CAT) and superoxide dismutase (SOD) activity, as well as the total antioxidant capacity (TAC). Patients with CAS displayed enhanced oxidative stress compared to control subjects, probably exceeding their antioxidant capacity. Interestingly, patients with CAS and T2DM displayed less oxidative stress, possibly due to the benefits of their pharmacological therapy (metformin). Thus, reducing oxidative stress or enhancing antioxidant capacity through specific therapies could be a good strategy to manage CAS, focusing on personalized medicine.

## 1. Introduction

Calcific aortic stenosis (CAS) is a chronic and progressive condition characterized by the thickening of the aortic valve (AV), which leads to diminished blood flow from the left ventricle into the aorta, ultimately provoking heart failure [1]. CAS is an active disease that involves lipoprotein deposition, chronic inflammation and, in advanced stages, the formation of calcium deposits in valvular tissue [2]. The pathophysiology of CAS is complex, and the only effective treatment to date is AV replacement [3]. Recent studies have suggested that when an individual is diagnosed with type 2 diabetes mellitus (T2DM), the progression from mild to severe CAS is accelerated and the prognosis of CAS becomes worse [4]. Furthermore, the prevalent comorbidities in individuals with T2DM and CAS—such as hypertension or dyslipidemia—further complicate the management of these patients [5,6].

Oxidative stress is one of the mechanisms involved in the initiation and progression of vascular tissue calcification [7]. Moreover, oxidative stress also contributes significantly to the development of macro- and microvascular complications in DM [8]. An imbalance between the production of reactive oxygen species (ROS) and the cellular antioxidant capacity of a tissue provokes oxidative damage, which generally has deleterious physiological consequences. ROS are essential mediators of biological activity that are generated as part of a cell’s metabolism. However, excessive production of ROS can exceed the cell’s antioxidant capacity and induce reversible or irreversible damage to macromolecules, including proteins, lipids, and DNA, ultimately provoking cell and tissue dysfunction [9]. ROS overproduction activates antioxidant enzymes, such as superoxide dismutase (SOD) or catalase, which scavenge the excess ROS. As it is difficult to quantify the ROS produced given their very short half-life, a common means to assess oxidative stress is to measure the oxidative modifications to macromolecules. Nevertheless, this approach is limited, as it frequently focuses on only one of a few markers of oxidative damage, without contemplating the antioxidant capacity, thereby failing to provide a complete view of oxidative status. Thus, it is necessary to measure multiple biomarkers of both oxidative damage and antioxidant defense to obtain a more global overview of oxidative stress [10].

A classic and widely accepted first-line therapeutic option for hyperglycemia in DM is the administration of metformin, currently considered the “gold standard” therapy for this condition. This drug stands out not only for its glycemic control, but also because it promotes several improvements in endothelial dysfunction, insulin resistance, and lipid profiles, as well as enhancing the performance of antioxidant systems [11]. These properties may mean that metformin offers some protection against the development of the macrovascular complications associated with DM, which was first suggested as early as 1995 [12]. Nevertheless, later meta-analyses have raised doubts about the effectiveness of metformin in reducing the risk of complications [12,13]. Recently, a meta-analysis suggested that the CV effects of metformin could be smaller than those reported by the United Kingdom Prospective Diabetes Study in 1995 [14]; however, this should be interpreted with caution, because there had only been a small number of randomized controlled trials to be included in the meta-analysis [15]. Another key point for controlling CV risk could be the anti-inflammatory effect of the drug, which suppresses the neutrophil-to-lymphocyte ratio—a systemic inflammation marker—as well as plasma cytokines [16]. Interestingly, there is also considerable evidence that metformin can inhibit oxidative stress through different pathways [17,18]. Moreover, it has been described that metformin exerts neuroprotective effects by regulating ischemic-stroke-induced oxidative stress injury [19]. Additionally, it has proven effective in ameliorating oxidative stress in several studies related to kidney disease [20]. However, to date, metformin’s effects have not been studied in the context of CAS.

Although several studies have demonstrated that oxidative stress plays important roles in the progression of CAS and T2DM individually, the global oxidative status of patients with both of these pathologies has yet to be evaluated. Moreover, the effect of metformin on oxidative stress has not been assessed in the context of CAS. Here, we adopted a multimarker approach to examine the global oxidative status of CAS patients, both with and without T2DM.

## 2. Materials and Methods

### 2.1. Study Population

**Figure 1:**
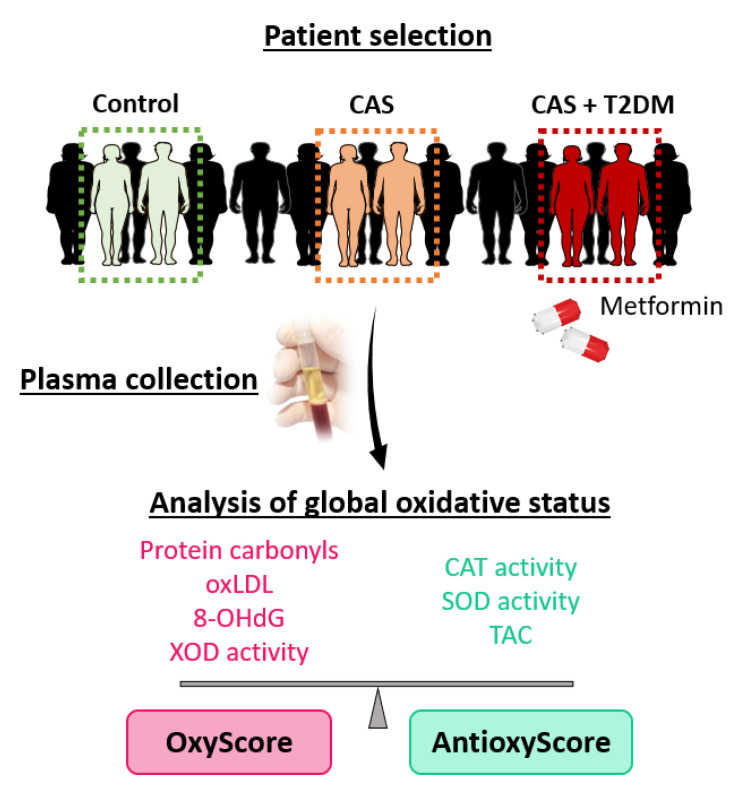
Experimental design. Plasma samples were collected and classified into three groups: control subjects, patients with calcific aortic stenosis (CAS), and patients with CAS and type 2 diabetes mellitus (T2DM) under metformin treatment. Oxidative damage and antioxidant defense biomarkers in the plasma were analyzed, and they were combined in multimarker scores of global oxidative damage (OxyScore) and antioxidant defense (AntioxyScore).

In this cross-sectional study, peripheral blood samples were collected from control subjects without CAS or T2DM and with two or fewer CV risk factors (*n* = 20), patients with CAS (*n* = 18), or patients with CAS and T2DM with metformin treatment (*n* = 18), all recruited at the Hospital General Universitario de Toledo (Spain) or the Hospital 12 de Octubre (Madrid, Spain). Any patient with a severe morbidity (e.g., ischemic heart disease with ventricular dysfunction, end-stage chronic kidney disease), bicuspid AV, a family or personal history of aortopathy, rheumatic valve disease, or moderate–severe mitral valve disease was excluded from the study. To assess the markers of oxidative damage and antioxidant defense, blood samples were collected in EDTA tubes and immediately centrifuged at 1125× *g* for 10 min, before being stored at −80 °C until use (Figure 1). 

This study was approved by the local ethics committee, and it was carried out in compliance with the tenets of the Helsinki Declaration. All patients signed an informed consent form prior to their inclusion in the study.

### 2.2. Biomarkers of Oxidative Damage

Oxidative damage to proteins, lipids, and DNA was determined through the presence of protein carbonyls, the oxidized low-density lipoprotein (oxLDL), and the 8-hydroxy-20-deoxyguanosine (8-OHdG) levels, respectively. Protein carbonyl groups were assayed using 2,4-dinitrophenylhydrazine through a protocol adapted for a microplate reader [21], and they were expressed as nmol/mg of total protein. OxLDL and 8-OHdG were assayed using commercial enzyme-linked immunosorbent assay (ELISA) kits (Mercodia AB, Uppsala, Sweden and Stress-MarqBiosciences Inc., Victoria, Canada, respectively), according to the manufacturer’s instructions. Pro-oxidant xanthine oxidase (XOD) activity was estimated with the Amplex Red assay (Invitrogen, Carlsbad, CA, USA) and expressed as mU/mg of total protein.

### 2.3. Biomarkers of Antioxidant Defense

The enzymatic antioxidant activity of plasma catalase was measured using the Amplex Red assay (Invitrogen) and expressed as U/mg of total protein. SOD activity was estimated with a colorimetric assay (Invitrogen) and expressed as mU/mg of total protein. The overall activity of low-molecular-weight antioxidants or the total antioxidant capacity (TAC) was determined using an assay based on enhanced horseradish-peroxidase-catalyzed luminol chemiluminescence adapted for a microplate reader [22]. Luminescence inhibition after the addition of plasma was used to calculate the area under the curve (AUC).

### 2.4. OxyScore and AntioxyScore

Oxidative damage and antioxidant defense biomarkers were combined in multimarker scores of global oxidative damage (OxyScore) and antioxidant defense (AntioxyScore), as described previously [23,24,25]. Briefly, markers of oxidative damage or antioxidant defense were standardized for each subject, using the healthy subjects as a reference. The sum of the standardized values for protein carbonyls, oxLDL, 8-OHdG, and XOD activity was used to calculate the OxyScore, whereas the sum of the standardized values of catalase and SOD activity, and of the TAC, was used to calculate the AntioxyScore.

### 2.5. Statistical Analysis

Statistical analyses were performed using GraphPad Prism 8 software (GraphPad Software Inc., San Diego, CA, USA) and SPSS 15.0 software for Windows (SPSS Inc., Chicago, IL, USA). First, normality was assessed with the Kolmogorov–Smirnov test; normally distributed variables were analyzed by parametric tests, and non-normally distributed variables were analyzed by non-parametric tests. Subsequently, differences in clinical parameters between groups were calculated by the chi-squared test in discrete variables and continuous variables, and oxidative markers were calculated by one-way ANOVA or Welch’s ANOVA and Bonferroni’s post hoc analysis in three-group comparisons, while Student’s *t*-test or the Mann–Whitney test was employed to calculate the differences between two groups. Pearson’s or Spearman’s correlation coefficients were calculated for the correlation analysis. The descriptive data were presented as the mean ± standard deviation (SD), or as percentages.

## 3. Results

The study was performed on a cohort of 56 patients divided into three study groups: control subjects, patients with CAS, and patients with CAS and T2DM under metformin treatment.

### 3.1. Clinical Characteristics

The clinical characteristics of the study groups are listed in Table 1. There were no significant differences between the groups in terms of the main cardiovascular risk factors, with the exceptions of cholesterol, HDL, glycemia, and metformin treatment. Patients with CAS, T2DM, and metformin treatment had lower levels of cholesterol and HDL in comparison with CAS patients without T2DM. HDL levels were also lower in patients with T2DM in comparison with control subjects. On the other hand, glycemia was higher in patients with CAS and T2DM in comparison with CAS patients without T2DM and control subjects. There were also no differences in Doppler echography data between CAS patients with and without T2DM. Differences in glycemia and metformin treatment were because of the presence of T2DM in one of the study groups. Inflammatory markers such as leukocyte counts or fibrinogen also showed no significant differences between the study groups.

To understand the differences in lipid profiles, and due to the relationship between metformin and lipids, we performed correlation analysis of them. The results showed that metformin treatment was negatively correlated with cholesterol (*r* = −0.353; *p*-value = 0.035) and HDL (*r* = −0.573; *p*-value = 0.000) (Table 2). On the other hand, inflammatory markers (leukocyte counts and fibrinogen) were not correlated with metformin treatment (Supplementary Table S1).

### 3.2. Markers of Oxidative Damage

**Figure 2:**
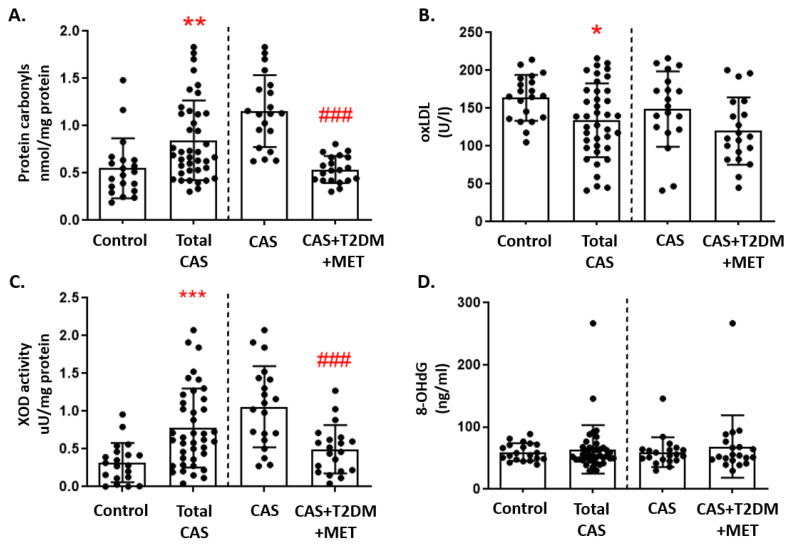
Markers of oxidative damage in CAS patients with and without T2DM: (**A**) Protein carbonyls, (**B**) oxidized LDL (oxLDL), (**C**) xanthine oxidase (XOD) activity, and (**D**) 8-hydroxy-2’-deoxyguanosine (8-OHdG) are represented as the mean ± SD. All of the graphs show the comparison between control subjects and total CAS patients (including CAS with T2DM and metformin treatment), and that between the CAS patients with and without T2DM, separated by a discontinuous line; * *p* < 0.05, ** *p* < 0.01, *** *p* < 0.001 control subjects vs. total CAS; ### *p* < 0.001 CAS vs. CAS + T2DM + MET.

Protein carbonylation is indicative of oxidative damage to proteins, and this was significantly higher in subjects with CAS than in control subjects (*p*-value < 0.001). Moreover, when CAS patients with and without T2DM were compared, patients with T2DM had significantly fewer carbonylated proteins (*p*-value < 0.001: Figure 2A). Oxidative damage to lipids was measured through the oxLDL levels, which were significantly lower in patients with CAS than in control subjects (*p*-value < 0.01). However, oxLDL was not significantly affected by T2DM, as CAS patients with and without T2DM had similar oxLDL values (Figure 2B). Moreover, there was significantly higher pro-oxidant XOD enzymatic activity in patients with CAS than in the group of control subjects (*p*-value < 0.001), although CAS patients with T2DM had significantly weaker XOD activity than CAS patients without T2DM (*p*-value < 0.001: Figure 2C). By contrast, the 8-OHdG levels—which served as a measure of oxidative damage to DNA—did not differ significantly between the three groups (Figure 2D). The remaining comparisons and statistical details are shown in Supplementary Table S2.

### 3.3. Markers of Antioxidant Defense

**Figure 3:**
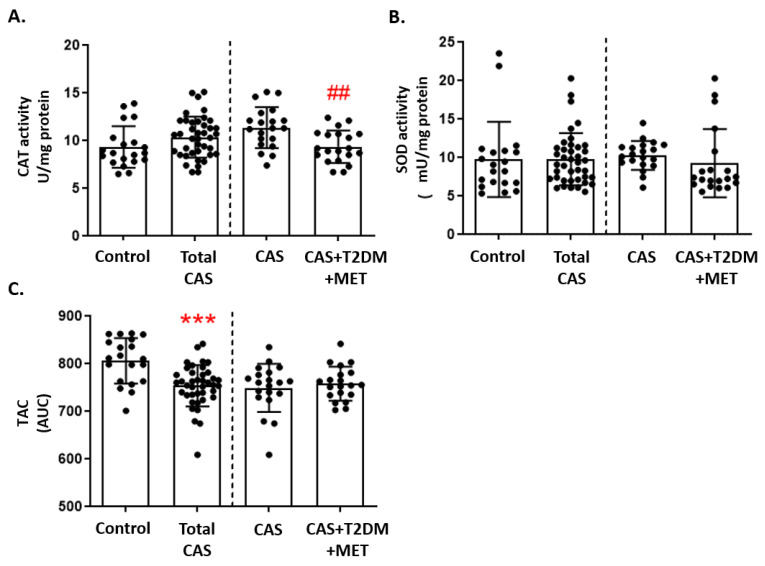
Markers of antioxidant defense in CAS patients with and without T2DM: (**A**) Catalase (CAT) activity, (**B**) superoxide dismutase (SOD) activity, and (**C**) total antioxidant capacity (TAC), quantified as the area under curve (AUC), are represented as the mean ± SD. The graphs show the comparison between the healthy control subjects and the total CAS patients (including CAS with T2DM and metformin treatment), and between CAS patients with and without T2DM, separated by a discontinuous line; *** *p* < 0.001 healthy control subjects vs. total CAS; ## *p* < 0.01 CAS vs. CAS + T2DM. MET: metformin.

Catalase activity did not differ between CAS patients and control subjects, although we did find a significant decrease in patients with CAS and T2DM relative to CAS alone (*p*-value < 0.01: Figure 3A). In contrast, there were no differences in SOD activity between the three groups (Figure 3B). Moreover, the luminescence in the TAC assay was significantly lower in CAS patients than in control subjects (*p*-value < 0.001: Figure 3C). The remaining comparisons and statistical details are shown in Supplementary Table S2.

### 3.4. Global Oxidative Status

**Figure 4:**
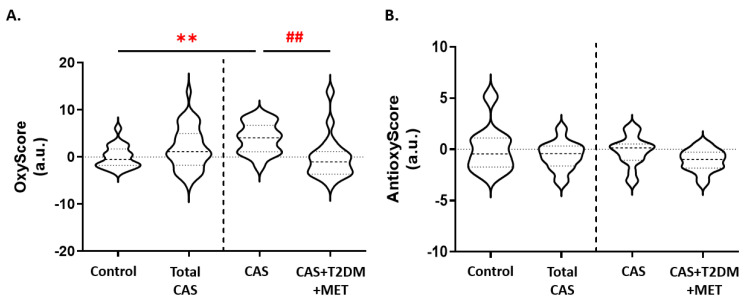
Variations in the (**A**) OxyScore and (**B**) AntioxyScore of CAS patients with and without T2DM. The graphs show the comparison between the control subjects and all of the patients with CAS (including CAS with T2DM and metformin treatment), and between CAS patients with and without T2DM, separated by a discontinuous line; ** *p* < 0.01 control subjects vs. CAS; ## *p* < 0.01 CAS vs. CAS + T2DM + MET. MET: metformin.

We found that the multimarker score of oxidative damage (OxyScore) was significantly higher in all patients with CAS than in control subjects (*p*-value < 0.01), whereas in CAS patients with T2DM the OxyScore was significantly lower than in those without T2DM (Figure 4A). In contrast, there were no differences in the multimarker antioxidant defense score (AntioxyScore) between the groups, although there was a non-significant trend towards a decrease in CAS patients with T2DM relative to CAS subjects without T2DM (Figure 4B). The remaining comparisons and statistical details are shown in Supplementary Table S2.

### 3.5. Metformin and Markers of Oxidative Stress and Inflammation

Correlation analysis between metformin treatment and markers of oxidative stress showed that metformin was negatively correlated with protein carbonylation (*r* = −0.28; *p*-value = 0.036), oxLDL levels (*r* = −0.35; *p*-value = 0.009), and OxyScore (*r* = −0.31; *p*-value = 0.022) (Table 3).

Regarding inflammatory markers, fibrinogen levels were not correlated with any oxidative stress marker (Supplementary Table S3). However, leukocyte count was positively correlated with catalase activity (*r* = 0.278; *p*-value = 0.040) and negatively correlated with total antioxidant capacity (*r* = −0.363; *p*-value = 0.006: Table 4).

## 4. Discussion

Personalized medicine is an emerging concept that involves managing the health of patients based on their individual characteristics, which could include features such as their particular oxidative status. As such, determining oxidative status may be useful to predict risk in cardiovascular diseases in general [23,26], and in CAS and T2DM in particular, as described here [7,27]. Indeed, oxidative stress contributes to the development of vascular complications in DM [28]; hence, patients with T2DM have a significant risk of CAS, and they are more likely to progress rapidly from mild to severe CAS [29]. ROS are important mediators of oxidative damage, and there is evidence of the importance of natural antioxidants in blocking the harmful effects of ROS and in halting the progression of CAS [30].

Given the association established between oxidative stress and the pathogenesis of CAS (and its relationship with T2DM), for the first time, we have evaluated the global oxidative status associated with CAS and T2DM, using the multimarker parameters OxyScore and AntioxyScore. These scores consider different aspects of oxidative damage and antioxidant defense, thereby offering a wider perspective of the variations in oxidative status in CAS and T2DM patients. In reference to DM, metformin has long been used to manage T2DM, and it remains the “gold standard” therapy for this condition, offering several benefits. Interestingly, there is considerable evidence that metformin can inhibit oxidative stress [17,18]—although, to date, its effects have not been studied in the context of CAS.

### 4.1. Oxidative Status in CAS Patients

Here, we found a positive association between CAS and oxidative damage to proteins, as assessed by protein carbonylation. We also found an association with lipid damage after the analysis of oxLDL and the pro-oxidant XOD activity, while oxidative damage to DNA—assessed through 8-OHdG—was not associated with CAS. The oxidation of protein backbones or the oxidative deamination of lysine or glutamic acid generates carbonyl groups, which are associated with aging and some CV risk factors [31]. This is consistent with the increase in protein carbonyls that we found in CAS patients. Additionally, the observed modifications are also positively correlated with age and smoking. Oxidative damage to proteins also induces the formation of protein aggregates and the loss of protein function [32,33]. Moreover, the increase in the number of carbonyl groups, as we observed in patients with CAS, has been previously reported to affect fibrinolysis in this kind of subject [34]. In addition, XOD catalyzes the oxidation of xanthine, as well as producing uric acid, hydrogen peroxide, and O_2_^−^ [33]. Experimental studies have demonstrated an increase in XOD activity in inflammation, diabetes, and cardiovascular disease (CVD) [35,36], which is entirely consistent with the increase in XOD activity that we observed in CAS patients relative to controls. On the other hand, oxLDL is the main promotor of the development of atherosclerosis, while also driving its progression and being directly associated with the risk of coronary artery disease [37]. Increased oxidative stress enhances the levels of oxLDL which, in turn, stimulates calcification [38]. Moreover, oxLDL contributes to the adherence and extravasation of immune cells through the expression of adhesion molecules [39]. Although an increase in oxLDL was expected in patients with CAS, lower levels of oxLDL were found, which we speculate may in part be due to the stringent control of risk factors for CVD in these patients, including cholesterol. Individually, the level of each marker is related to different processes, all of which are important for the development of CVD in a different manner. 

Altogether, the sum of the oxidative markers—including oxLDL and 8-OHdG—resulted in an OxyScore that was significantly higher in patients with CAS. Interestingly, measurement of oxLDL or 8-OHdG alone would lead to the opposite result, which underlines the importance of multimarker analysis to evaluate global oxidative stress. In terms of markers of antioxidant defense, the AntioxyScore was the sum of the contributing factors, yet it did not differ between the groups. Oxidative stress was originally defined as an increase in ROS production and a decrease in antioxidant defense mechanisms [40]. However, it has recently been demonstrated that oxidative stress is the main activator of antioxidant defense mechanisms—for example, through the activity of the highly conserved transcription factor Nrf2 [41]. These factors may regulate the transcription of different cytoprotective genes. Our results indicate that despite the increase in oxidative end-products, CAS patients do not respond properly to the oxidative insult; that is, the mechanisms involved in the activation of the antioxidant defenses may be altered. Therefore, reducing oxidative stress through personalized therapies in patients with CAS should be considered to improve their management. Additionally, it would also be interesting to deepen our knowledge of these impaired mechanisms in order to develop new treatments for these patients.

### 4.2. Oxidative Status in CAS Patients with T2DM

Oxidative stress in T2DM is both a cause and a consequence of the disease. The pancreatic β-cells that produce and secrete insulin are vulnerable to the damaging effects of oxidative stress due to their impaired antioxidant defense. This, in turn, leads to β-cell dysfunction and death, activating pathways that are linked to insulin resistance [42]. In addition, hyperglycemia increases protein glycation at protein carbonyl groups, which trigger the generation of ROS and nitric oxide, worsening the pro-oxidant state [43]. Therefore, DM is associated with markers of lipid oxidation and of DNA/RNA and protein damage, as well as defects in the antioxidant systems [44]. Consequently, we expected an increase in the OxyScore for patients with CAS and T2DM relative to those without T2DM. However, CAS patients who were treated for T2DM had significantly fewer carbonyl groups and lower XOD activity than CAS patients without T2DM. Moreover, the global analysis of OxyScore also showed significant differences between the groups. Conversely, while we found a significant decrease in two antioxidant markers—TAC and catalase activity—the AntioxyScore did not show significant differences between T2DM and non-T2DM patients. Weaker TAC and catalase activity occurred concomitant to the decrease in oxidative damage in T2DM patients. 

### 4.3. Metformin, Oxidative Stress, and Inflammation

We also found a negative correlation between metformin treatment and carbonyl groups, oxLDL, OxyScore, and cholesterol or HDL levels. Metformin reduces blood glucose through several mechanisms, including the reduction in glucagon secretion and glucose absorption during digestion, and an increase in peripheral glucose uptake [45]. Moreover, several studies have assigned an anti-inflammatory, anti-apoptotic, anti-atherogenic, and antioxidant role to metformin [46,47,48]. The results of correlation analysis could explain why CAS patients with T2DM under metformin treatment have better lipid profiles and once again demonstrate the benefit of metformin with respect to lipid profiles. Furthermore, these results could also explain why oxLDL levels were lower in the total CAS population vs. controls. There is also evidence that the primary site of action of metformin is through the direct inhibition of mitochondrial complex 1, which contributes to ROS production [49,50]. When this complex is inhibited, ROS production decreases [51,52], which suggests that metformin decreases mitochondrial ROS levels. Similarly, studies on subjects with T2DM [53] and on animal models [54] showed that metformin decreases ROS and reactive nitrogen species, thereby restoring antioxidant status. Recent experimental studies [55] in human umbilical vein endothelial cells, in vitro and in vivo, demonstrated that metformin exerts its protective effects against cell damage by inhibiting the release of malondialdehyde (MDA)—a marker of lipid peroxidation. These findings indicate that ROS generation triggered by methylglyoxal (MGO)—a glycolysis side-product—is blocked by metformin, and they support the hypothesis that metformin prevents MGO-induced apoptosis through its protective effect on mitochondrial function. Indeed, metformin effectively offers protection against MGO-induced oxidative stress, mitochondrial dysfunction, apoptosis, and inflammation in vitro and in vivo [55]. Specifically, metformin prevents the apoptotic signaling cascades initiated by MGO-generated ROS by modulating PI3K/Akt and Nrf2/HO-1 signaling. This compelling evidence expands our understanding of the benefits and clinical applications of metformin therapy, providing novel insights for the development of strategies to preserve endothelial function in diabetic vascular diseases.

Inflammation has been also associated with oxidative stress and metformin [11]. However, we did not find a correlation between metformin treatment and inflammatory markers in our population. Instead, leukocyte counts were positively correlated with catalase activity and negatively correlated with TAC. This dual correlation—positive and negative—does not clarify the global effect. Moreover, we did not find differences in the multimarker antioxidant defense AntioxyScore (which involves both markers) between groups.

The main limitations of this study were the small cohort and different comorbidities. Moreover, factors such as obesity, sedentariness, or diet may—more or less—affect the oxidative status of the groups enrolled [56,57]. Thus, further studies with larger cohorts are needed to confirm the beneficial effect of metformin in diabetic patients with CAS. Moreover, by increasing the number of patients, it would also be possible to adequately stratify patients by age, comorbidities, or treatments, according to these multimarker scores. This will be an important step to improve personalized medicine and offer an adequate treatment to each patient, improving the management by clinicians. The results obtained here demonstrate the antioxidant effect of metformin. In diabetic patients, treatment with metformin and the strict control of CVD risk factors achieves good results in terms of oxidative stress. Moreover, a recent study in a cell model reported that metformin can inhibit calcification of the AV interstitial cells by activating PI3K/AKT signaling [58]. All of these findings indicate that metformin therapy is a good strategy to improve the clinical management of patients with CAS and T2DM—although, to date, it does not appear to be sufficient to avoid CAS progression. It would be interesting to have a group of patients with CAS and T2DM without metformin treatment to analyze the sole impact of T2DM. Current clinical management guidelines for the management of diabetes recommend the use of metformin as the first-line treatment for T2DM [59]. Therefore, only newly diagnosed patients would be included in this group, which is beyond the scope of this study, as it focuses on the role of T2DM in the development of CAS. Future studies may study the early stages of the disease, such as valve sclerosis, which could include newly diagnosed T2DM patients, i.e., without treatment.

## 5. Conclusions

**Figure 5:**
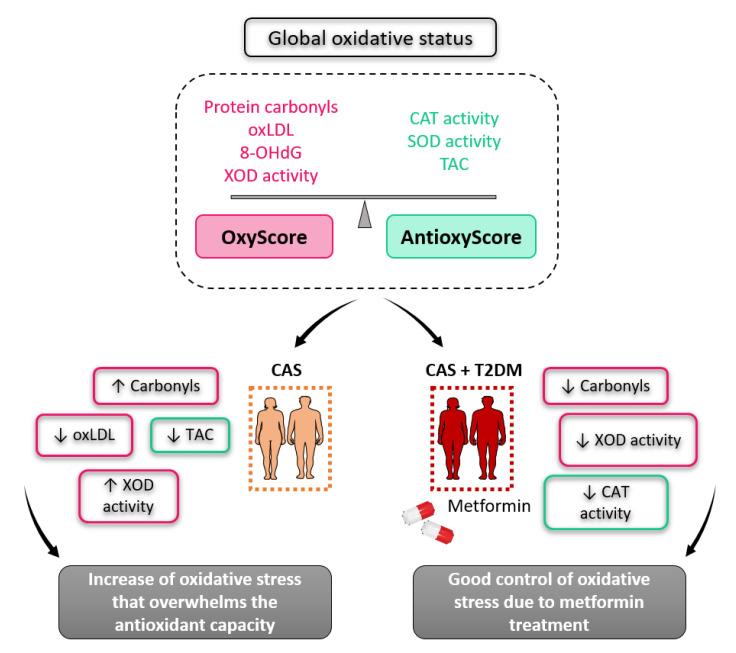
Schematic overview of the global oxidative status in patients with calcific aortic stenosis, with and without type 2 diabetes mellitus.

In conclusion, oxidative stress increases in CAS patients, and it probably overwhelms their antioxidant capacity relative to control subjects, while patients with CAS and T2DM have less oxidative stress compared to those without T2DM—probably due to the benefits of metformin (Figure 5). Our results enforce the multifaceted role of metformin, which has different mechanisms of action that have been revealed one after another in its long history. Therefore, introducing personalized therapies for CAS patients that reduce oxidative stress or supplement antioxidants could be a good strategy to manage CAS and enhance their antioxidant capacity.

## Figures and Tables

**Table 1:** Clinical characteristics of the subjects recruited. The values are expressed as the percentages (%) or mean ± standard deviation; # *p* < 0.05, ### *p* < 0.001 CAS vs. CAS + T2DM + metformin; †† *p* < 0.01, ††† *p* < 0.001 control subjects vs. CAS + T2DM + metformin. HDL, high-density lipoprotein; LDL, low-density lipoprotein; AV, aortic valve.

	Control (*n* = 20)	CAS (*n* = 18)	CAS + T2DM + Metformin (*n* = 18)	*p*-Value
Age (years)	71 ± 9	76 ± 6	72 ± 9	0.21
Sex (male) (%)	50	50	50	1.00
Obesity (%)	25	17	28	0.71
Hypertension (%)	90	72	89	0.26
Dyslipidemia (%)	45	67	50	0.38
Smoker (%)	10	28	11	0.26
Cholesterol (mg/dL)	168 ± 46	164 ± 24	137 ± 36 #	0.03
HDL (mg/dL)	55 ± 15	53 ± 18	39 ± 9 #,††	0.00
LDL (mg/dL)	91 ± 36	92 ± 19	76 ± 31	0.20
Triglycerides (mg/dL)	106 ± 41	94 ± 33	110 ± 49	0.44
Glycemia (mg/dL)	96 ± 9	100 ± 11	139 ± 43 ###,†††	0.00
Leukocytes (×10^9^/mL)	7.0 ± 2	8.0 ± 2	7.7 ± 2	0.30
Fibrinogen (mg/dL)		441 ± 99	480 ± 122	0.34
AV area (cm^2^)	-	0.82 ± 0.14	0.85 ± 0.16	0.60
Ejection fraction (%)	-	59 ± 13	57 ± 13	0.61
Mean gradient (mmHg)	-	41 ± 16	39 ± 17	0.69
Peak gradient (mmHg)	-	66 ± 25	74 ± 28	0.45
Systolic diameter (mm)	-	27 ± 4	30 ± 6	0.12
Diastolic diameter (mm)	-	44 ± 6	45 ± 6	0.55
Septum (mm)	-	14 ± 2	14 ± 2	0.47
Drugs				
Anti-hypertensives (%)	85	83	94	0.55
Lipid-lowering agents (%)	65	56	61	0.84
Antidiabetic agent (%) (metformin)	0	0	100	0.00

**Table 2:** Correlation of metformin treatment with lipid profile. HDL, high-density lipoprotein; LDL, low-density lipoprotein; *r*, pearson’s coefficient. * *p* < 0.05, ** *p* < 0.01.

	*r*	*p*-Value
Cholesterol *	−0.353	0.035
HDL **	−0.573	0.000
LDL	−0.225	0.118
Triglycerides	−0.088	0.609

**Table 3:** Correlation of metformin treatment with markers of oxidative damage and antioxidant defense: oxLDL, oxidized low-density lipoprotein; 8-OHdG, 8-hydroxy-2’-deoxyguanosine; XOD, xanthine oxidase; SOD, superoxide dismutase; TAC, total antioxidant capacity. * *p* < 0.05, ** *p* < 0.01.

	*r*	*p*-Value
Protein carbonyls *	−0.279	0.036
oxLDL **	−0.348	0.009
8-OHdG	−0.044	0.746
XOD activity	−0.138	0.308
Catalase activity	−0.062	0.652
SOD activity	−0.023	0.866
TAC	−0.174	0.199
OxyScore *	−0.306	0.022
AntioxyScore	−0.153	0.261

**Table 4:** Correlation of leukocyte count with oxidative stress markers. * *p* < 0.05, ** *p* < 0.01.

	*r*	*p*-Value
Protein carbonyls	0.083	0.548
oxLDL	0.104	0.454
XOD activity	0.103	0.455
8-OHdG	0.079	0.566
Catalase activity *	0.278	0.040
SOD activity	−0.116	0.399
TAC **	−0.363	0.006
OxyScore	0.185	0.177
AntioxyScore	−0.102	0.457

## Data Availability

The data presented in this study are available in the article and Supplementary Materials.

## Supplementary Materials

The following supporting information can be downloaded at: https://www.mdpi.com/article/10.3390/antiox12051024/s1, Table S1: Correlation of inflammatory markers with metformin treatment; Table S2: Statistical differences of oxidative damage and antioxidant defense markers. *p*-values from ANOVA *t*-test and Bonferroni post hoc are shown. C: control; CAS: calcific aortic stenosis; T2DM: diabetes mellitus type 2; Ns: non-significant; MET: metformin; Table S3: Correlation of fibrinogen levels with oxidative stress markers.
