# Supplementary material for: Global Oxidative Status Is Linked to Calcific Aortic Stenosis: The Differences Due to Diabetes Mellitus and the Effects of Metformin
Source: Antioxidants (Basel). 2023 Apr 28;12(5):1024. doi: 10.3390/antiox12051024 (PMC10215415; doi:10.3390/antiox12051024)
Supplement: Supplementary file 1 [file antioxidants-12-01024-s001.zip › antioxidants-2288685-supplementary.pdf]

Supplementary Material

Global Oxidative Status Is Linked to Calcific Aortic Stenosis: The Differences due to Diabetes Mellitus and the Effects of Metformin

Nerea Corbacho-Alonso [1,2,†], Elena Rodríguez-Sánchez [3,†], Tamara Sastre-Oliva [1,2], Elisa Mercado-García [3], Ines Perales-Sánchez [1,2], Cristina Juarez-Alia [1,2], Luis F. López-Almodovar [4], Luis R. Padial [5], Teresa Tejerina [6], Laura Mourino-Alvarez [1,2,‡], Gema Ruiz-Hurtado [3,7,*,‡] and María G. Barderas [1,2,*,‡]

[1] Department of Vascular Physiopathology, Hospital Nacional de Paraplejicos, SESCAM, (Servicio de Salud de Castilla-La Mancha), 45071 Toledo, Spain
[2] Department of Vascular Physiopathology, Hospital Nacional de Paraplejicos, Instituto de Investigación Sanitaria de Castilla-La Mancha (IDISCAM), 45071 Toledo, Spain
[3] Cardiorenal Translational Laboratory, Instituto de Investigación Imas12, Hospital Universitario 12 de Octubre, 28041 Madrid, Spain
[4] Cardiac Surgery, Hospital General Universitario de Toledo, SESCAM, 45007 Toledo, Spain
[5] Department of Cardiology, Hospital General Universitario de Toledo, SESCAM, 45007 Toledo, Spain
[6] Department of Pharmacology, School of Medicine, Universidad Complutense de Madrid, 28040 Madrid, Spain;
[7] Centro de Investigación Biomédica en Red de Enfermedades Cardiovasculares, CIBER-CV Hospital Universitario 12 de Octubre, 28041 Madrid, Spain

* Correspondence: gemaruiz@h12o.es (G.R.-H.); megonzalezb@sescam.jccm.es or marucitos@hotmail.com (M.G.B.); Tel.: +34-91-390-8001 (G.R.-H.); +34-925396826 (M.G.B.)

† These authors contributed equally to this work.

‡ These authors contributed equally to this work.

Table S1. Correlation of inflammatory markers with metformin treatment.

	r	*p*-value
**Leukocyte count**	0.05	0.693
**Fibrinogen**	0.18	0.337

Table S2. Statistical differences of oxidative damage and antioxidant defense markers. *p*-values from ANOVA *t*-test and Bonferroni post hoc are shown. C: control; CAS: calcific aortic stenosis; T2DM: diabetes mellitus type 2; Ns: non-significant; MET: metformin.

	ANOVA	C vs Total CAS	C vs CAS + T2DM + MET	CAS vs CAS + T2DM + MET
**Protein carbonyls**	0.000	0.000	ns	0.000
**oxLDL**	0.005	ns	0.004	0.216
**8-OHdG**	0.732	ns	ns	ns
**XOD activity**	0.000	0.000	ns	0.000
**Catalase activity**	0.001	0.003	ns	0.006
**SOD activity**	0.593	ns	ns	ns
**TAC**	0.000	0.000	0.002	ns
**OxyScore**	0.000	0.002	ns	0.001
**AntioxyScore**	0.088	ns	ns	ns

Table S3. Correlation of fibrinogen levels with oxidative stress markers.

	r	*p*-value
**Protein carbonyls**	−0.149	0.425
**oxLDL**	−0.009	0.454
**8-OHdG**	−0.081	0.665
**XOD activity**	−0.253	0.170
**Catalase activity**	−0.117	0.532
**SOD activity**	−0.111	0.552
**TAC**	0.014	0.939
**OxyScore**	−0.231	0.211
**AntioxyScore**	−0.129	0.488
